# Epidemiology and clinical manifestations of reported Lyme disease cases: Data from the Canadian Lyme disease enhanced surveillance system

**DOI:** 10.1371/journal.pone.0295909

**Published:** 2023-12-15

**Authors:** Kiera Murison, Christy H. Wilson, Katie M. Clow, Salima Gasmi, Todd F. Hatchette, Annie-Claude Bourgeois, Gerald A. Evans, Jules K. Koffi

**Affiliations:** 1 Infectious Diseases and Vaccination Programs Branch, Public Health Agency of Canada, Ottawa, Ontario, Canada; 2 Department of Population Medicine, University of Guelph, Guelph, Ontario, Canada; 3 Infectious Diseases and Vaccination Programs Branch, Public Health Agency of Canada, Saint-Hyacinthe, Québec, Canada; 4 Department of Pathology and Laboratory Medicine, Nova Scotia Health Authority, Departments of Pathology, Immunology and Microbiology, Medicine, Dalhousie University, Halifax, Nova Scotia, Canada; 5 Infection Prevention & Control, Kingston Health Sciences Centre, Biomedical & Molecular Sciences and Pathology & Molecular Medicine, Queen’s University, Kingston, Ontario, Canada; University of Bologna / Romagna Local Health Authority, ITALY

## Abstract

Lyme disease cases reported in seven Canadian provinces from 2009 to 2019 through the Lyme Disease Enhanced Surveillance System are described herein by demographic, geography, time and season. The proportion of males was greater than females. Bimodal peaks in incidence were observed in children and older adults (≥60 years of age) for all clinical signs except cardiac manifestations, which were more evenly distributed across age groups. Proportions of disease stages varied between provinces: Atlantic provinces reported mainly early Lyme disease, while Ontario reported equal proportions of early and late-stage Lyme disease. Early Lyme disease cases were mainly reported between May through November, whereas late Lyme disease were reported in December through April. Increased awareness over time may have contributed to a decrease in the proportion of cases reporting late disseminated Lyme disease. These analyses help better describe clinical features of reported Lyme disease cases in Canada.

## Introduction

Lyme disease (LD) is caused by infection with the tick-borne bacteria *Borrelia burgdorferi*, resulting in multi-system disease. It is clinically characterized by three disease stages depending on the level of dissemination of the infection: early localized, early disseminated, and late disseminated [1]. Early localized LD typically manifests on the skin as a single erythema migrans (EM) at the site of infection (i.e., location of tick bite) [2]. If the disease is not treated, it can disseminate to several organs leading to the development of early disseminated LD. This stage is reported in 12%–15% of individuals infected with *B*. *burgdorferi* [3–5], although there are reports as high as 40% [6]. The most frequent clinical signs of this phase are multiple EM and neurological manifestations such as aseptic meningitis, cranial neuropathy, and motor or sensory radiculopathy. Cardiac manifestations can accompany early disseminated LD as well in about 1% of individuals [7]. If the disease remains untreated, it can evolve months to years post-infection to the late disseminated phase that is characterised by possible musculoskeletal, neurological and cognitive manifestations. About 50% of those patients will develop Lyme arthritis that consists of intermittent episodes of pain and swelling in one or multiple joints, particularly the knees and other large joints [8].

In North America, *B*. *burgdorferi* is transmitted via the bite of a tick of the genus *Ixodes; Ixodes scapularis* and *Ixodes pacificus*. Northward range expansion of these ticks has been observed throughout North America, including parts of Canada [9]. Lyme disease is an emerging disease in Canada, with an increase from 144 reported cases in 2009 to 2,636 reported cases in 2019 [10]. To date, national surveillance reports have focused on reporting national LD incidence, cases by age-group, geographic distribution, and seasonality. Studies in Canada [10–12] and the Canadian province of Nova Scotia [13] have described demographics, disease stage risk factors and the proportions of LD cases reporting characteristic clinical manifestations. A recent study in Ontario examined the relationships between LD clinical manifestations and disease stages with seasonality, geography, demographics, and trends over time, providing insight at the regional level in the province [6]. This study aims to describe the clinical characteristics of LD cases acquired in Canada and reported through the Lyme Disease Enhanced Surveillance System by age group, disease stage and spatiotemporal factors.

## Materials and methods

### Data sources

LD cases are reported to the Public Health Agency of Canada (PHAC) by the 10 provinces and the 3 territories through the Canadian Notifiable Disease Surveillance System (CNDSS) and Lyme Disease Enhanced Surveillance (LDES) System of the PHAC as described elsewhere [11, 12, 14]. Provincial and territorial public health organizations are responsible for case management, including the classification of cases according to national case definition in use at the time of notification. The CNDSS allows for the collection of basic demographic and temporal information. The LDES system captures additional information, including clinical manifestations and location of acquisition. Seven provinces that participated in the LDES system as of 2019 were included in this analysis: British Columbia, Alberta, Ontario, New Brunswick, Nova Scotia, Prince Edward Island, and Newfoundland and Labrador. Manitoba participated in the LDES but was excluded from this analysis. Quebec submitted data to the LDES but did not provide clinical manifestations data and location of acquisition within the province, and thus was excluded from this analysis. Saskatchewan and the Territories did not submit data to the LDES and alternatively reported data to the CNDSS. Cases were classified as ‘confirmed’ or ‘probable’ by provincial public health organizations submitting the data, using the 2009 case definition for the period 2009–2015 [15] and the 2016 case definition for the period 2016–2019 [16] (Table 1). Together these two systems form the national surveillance system for Lyme disease. All information transferred to PHAC by the provinces and territories through these two systems are de-identified data.

**Table 1:** Lyme disease 2009 and 2016 case definitions, Canada.

2009 case definition [15]	2016 case definition [16]
**Confirmed case**	
**Clinical evidence of illness with laboratory confirmation**: • isolation of *Borrelia burgdorferi* from an appropriate clinical specimen OR • detection of *B*. *burgdorferi* DNA by PCR **OR** **Clinical evidence of illness with a history of residence in, or visit to, an endemic area** and with laboratory evidence of infection, i.e., positive serologic test using the two-tier* ELISA and Western Blot criteria	**Clinical evidence of illness with laboratory confirmation by one of the following methods**: • isolation of *Borrelia burgdorferi* from a clinical specimen as specified by current guidelines **OR** • detection of *B*. *burgdorferi* DNA by PCR testing on synovial fluid, cerebrospinal fluid, erythema migrans tissue biopsies or blood and by methods specified by current guidelines **OR** **Clinical evidence of illness with a history of residence in, or visit to, a Lyme disease risk area**; and with laboratory evidence of infection in the form of a positive serologic test using the Standard Two Tiers Testing algorithm*
**Probable case**	
**Clinical evidence of illness with a positive serologic test** result using the two-tier (ELISA and Western Blot) test, without a history of residence in, or visit to, a Lyme disease endemic area. **OR** **Clinician-observed erythema migrans without laboratory evidence** but with history of residence in, or visit to, a Lyme disease endemic area.	**Clinical evidence of illness without a history of residence in, or visit to, a Lyme disease risk area**; and with laboratory evidence of infection in the form of a positive serologic test as defined under confirmed cases. **OR** **Clinician-observed erythema migrans without laboratory evidence** but with history of residence in, or visit to, a Lyme disease risk area.

Abbreviations: DNA, deoxyribonucleic acid; ELISA, enzyme-linked immunosorbent assay; PCR, polymerase chain reaction

* Although some jurisdictions have started using the Modified Two-Tier Testing (MTTT) algorithm using two enzyme immunoassays (EIAs), these data were generated at a time when all laboratories in Canada were using the Standard Two Tier Testing algorithm (STTT) consisting of a screening ELISA followed by an immunoblot assay. Immunoblots include traditional Western blots or newer line blots, and both formats target an identical set of *B*. *burgdorferi* immune-reactive protein.

### Inclusion criteria

Cases used in the analysis include those reported to the LDES system with episode date between 2009 and 2019 which met the definition for a probable or confirmed case (Table 1), for which at least one clinical manifestation is recorded and with known locality of acquisition being in Canada. Cases reported by clinicians to regional public health organizations were investigated to determine if the patient lived in or had travelled to a Lyme disease risk area (also called Lyme disease endemic area from 2009–2015) within Canada in the past month. The probable locality of acquisition was determined from the case investigation. Any clinical manifestations were either recorded by the physician or self-reported by the patient as part of the investigation that follows the physician report. Clinical manifestations grouped as syndrome (EM, neurologic, cardiac or arthritis) are captured through a report form by provincial public health organizations and then transferred electronically to PHAC. Cases were then grouped by disease stage, accounting for the furthest progression of disease they displayed. Early localized cases were characterized as presenting with single EM only. Early disseminated cases were characterized by any of the following manifestations, with or without single EM: multiple EM, Bell’s palsy, other neurological symptoms, or cardiac manifestations (Lyme carditis). Late disseminated cases were characterized as any case presenting with recurrent joint swelling (Lyme arthritis), with or without reporting any other manifestations. For example, a case presenting with single EM and Bell’s palsy would be counted for both clinical manifestations but classified as early disseminated.

### Statistical analyses

Cumulative incidence per 100,000 population was calculated using Canadian census estimates [17] at the midpoint of the surveillance period, July 1, 2014. This denominator was used to address the dynamic nature of the Canadian population across the time period [18]. Mean annual incidence was calculated using population estimates on July 1^st^ for each year for the region indicated [17]. All geographic analyses only included LD cases that were acquired within the same province in which they were reported (locally acquired). As there were fewer than 5 locally-acquired cases in British Columbia, Alberta, Prince Edward Island, and Newfoundland and Labrador, these four provinces were excluded from geographic analyses. LD cases reported by Nova Scotia but without a location of acquisition were assumed to be acquired within the province, which is entirely an LD risk area. To account for small provincial counts, provinces were grouped into regions: Central if the province of acquisition was Ontario, and Atlantic if the province was acquisition was New Brunswick or Nova Scotia.

Trends over time were analyzed by comparing the proportion of LD cases reporting each symptom and disease stage between the years before (2009–2015) and after (2016–2019) the LD case definition was updated. The national surveillance case definition for LD was developed from 2007–2009 and LD became notifiable in 2009 [15]. In 2016, the case definition was revised to include five methods to identify risk areas. This approach allowed for more flexibility in identifying risk that accounts for differences in the geographic scope of LD risk in different jurisdictions and ensure consistency of reporting across Canada [16]. To temporally categorize cases, the reported date of symptom onset was used.

Standard deviation was calculated for monthly distribution of LD cases. Proportions were calculated with 95% confidence intervals (CI) using R (version 4.0.2). The Wilcoxon rank sum test was used to compare the difference in age between time periods. A two-tailed, two-sample t-test was used to compare the difference in mean annual incidences between time periods. Statistical significance was indicated by p<0.05. The geographic distribution of locally-acquired LD cases by stage was mapped using QGIS (version 3.8.1).

Logistic regression models were used to explore variations among region acquired, age group, sex, seasonality, and time period. In separate models, the outcome was the presence or absence of early localized, early disseminated, and late disseminated LD. For each model, explanatory variables were region, age group, sex, seasonality and whether the case was reported in the period before (2009–2015) or after (2016–2019) the LD case definition was updated. Only LD cases with no missing data were included in the model, and as with other geographic analyses, only locally-acquired cases were included using the regions defined above. For seasonality, month of reported date of symptom onset was used to create four categories. The reference groups for region, age group, sex, season, and time period were Atlantic, 0–9 years, female, December-February, and 2009–2015, respectively. The significance level for explanatory variables retained in the multivariable model was less than 0.1. The most parsimonious multivariate models were sought by backward elimination of nonsignificant variables until all factors in the model were significant (p<0.05).

### Ethics statement

Lyme disease enhanced surveillance data are routinely collected for surveillance and epidemiologic purposes and the extract used for this study did not include personal identifiers. As a result, ethics approval was not required.

## Results

### Case characteristics

**Fig 1:**
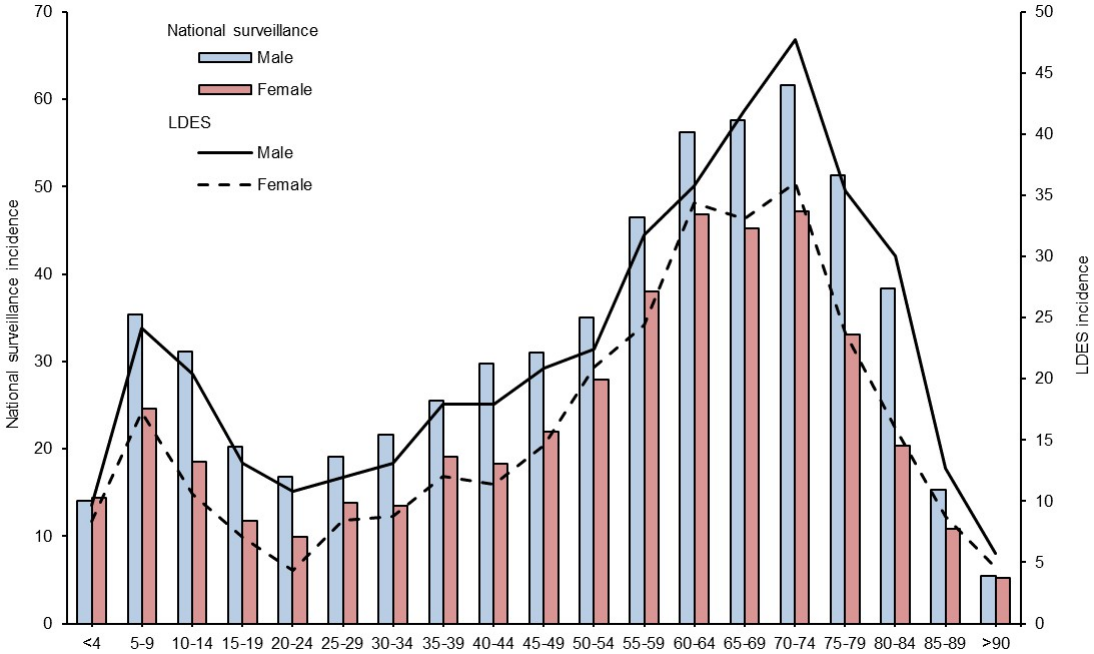
Comparison of age and sex distribution between national surveillance and the LDES system, 2009–2019. All Lyme disease cases in Canada (n = 9,969) and in the LDES subset (n = 4,690) with available data on age and sex were included. Incidence is per 100,000. The denominator in the LDES subset was the 7 provinces participating in the LDES with symptom data available for this study.

From 2009 to 2019, there were 10,150 LD cases reported to PHAC. Of these, 9,526 (93.9%) were acquired in Canada, and among those cases 4,701 (46.3%) reported at least one clinical manifestation which formed our dataset. Over two thirds of cases were confirmed (70.0%, 95% CI: 68.7–71.3%) (Table 2). The median age was 53 years (interquartile range [IQR]: 32–65) and 56.9% of cases were male (95% CI: 55.5–58.4%). For comparison, the median age of all LD patients submitted through the national surveillance system during the study period was 52 years (IQR: 31–64), and 57.3% of the population was male (95% CI: 56.3–58.2%) (Fig 1). Single EM was the most reported clinical manifestation (76.4%, 95% CI: 75.1–77.6), followed by Lyme arthritis (32.2%, 95% CI: 30.9–33.6).

**Table 2:** Characteristics of Lyme disease cases by clinical manifestations and by period of time, Canada, 2009–2019*.

Characteristic	All years (n = 4701)		2009–2015 (n = 1367)		2016–2019 (n = 3334)	
Age, median (IQR)	53 (32–65)		51 (30–63) *		54 (34–65) *	
Case classification, No./total, % (95% CI)						
Probable	1411/4701	30.0 (28.7–31.3)	447/1367	32.7 (30.2–35.3)	964/3334	28.9 (27.4–30.5)
Confirmed	3290/4701	70.0 (68.7–71.3)	920/1367	67.3 (64.7–69.8)	2370/3334	71.1 (69.5–72.6)
Sex, No./total, % (95% CI)						
Female	2022/4695	43.1 (41.6–44.5)	586/1365	42.9 (40.3–45.6)	1436/3330	43.1 (41.4–44.8)
Male	2673/4695	56.9 (55.5–58.4)	779/1365	57.1 (54.4–59.7)	1894/3330	56.9 (55.2–58.6)
Clinical manifestation**, No. /total, % (95% CI)						
Single EM	3588/4697	76.4 (75.1–77.6)	1023/1367	74.8 (72.4–77.1)	2565/3330	77.0 (75.6–78.4)
Multiple EM	309/1583	19.5 (17.6–21.6)	95/559	17.0 (14.0–20.4)	214/1024	20.9 (18.4–23.5)
Bell’s palsy	320/4313	7.4 (6.7–8.2)	109/1367	8.0 (6.6–9.5)	211/2946	7.2 (6.3–8.2)
Other neurological manifestation	858/4697	18.3 (17.2–19.4)	199/1367	14.6 (12.7–16.5)	659/3330	19.8 (18.4–21.2)
Lyme carditis	156/4691	3.3 (2.8–3.9)	48/1367	3.5 (2.6–4.6)	108/3324	3.2 (2.7–3.9)
Lyme arthritis	1511/4693	32.2 (30.9–33.6)	490/1367	35.8 (33.3–38.5)	1021/3326	30.7 (29.1–32.3)
Disease stage, No./total, % (95% CI)						
Early localized***	2339/4701	49.8 (48.3–51.2)	638/1367	46.7 (44.0–49.4)	1701/3334	51.0 (49.3–52.7)
Early disseminated****	851/4701	18.1 (17.0–19.2)	239/1367	17.5 (15.5–19.6)	612/3334	18.4 (17.1–19.7)
Late disseminated	1511/4701	32.1 (30.8–33.5)	490/1367	35.9 (33.3–38.5)	1021/3334	30.6 (29.1–32.2)

Abbreviation: IQR, interquartile range; CI, confidence interval; EM, erythema migrans.

* p<0.001

** Total numbers of cases with a clinical manifestation do not equal the total number of cases as a patient can report several symptoms (like single EM + Bell’s palsy). Proportions represent how many cases reported that symptom where data was available, regardless of disease stage.

*** The number of cases that reported single EM does not equal the number of cases diagnosed at early localized stage because some of these patients have been diagnosed at later stage of the disease.

**** The number of cases that reported multiple EM, Bell’s palsy, other neurological manifestation and Lyme carditis is different from the number of cases diagnosed at early disseminated stage because a patient can present several symptoms and some of these patients could be diagnosed at later stage of the disease.

### Incidence of clinical manifestations by age

**Fig 2:**
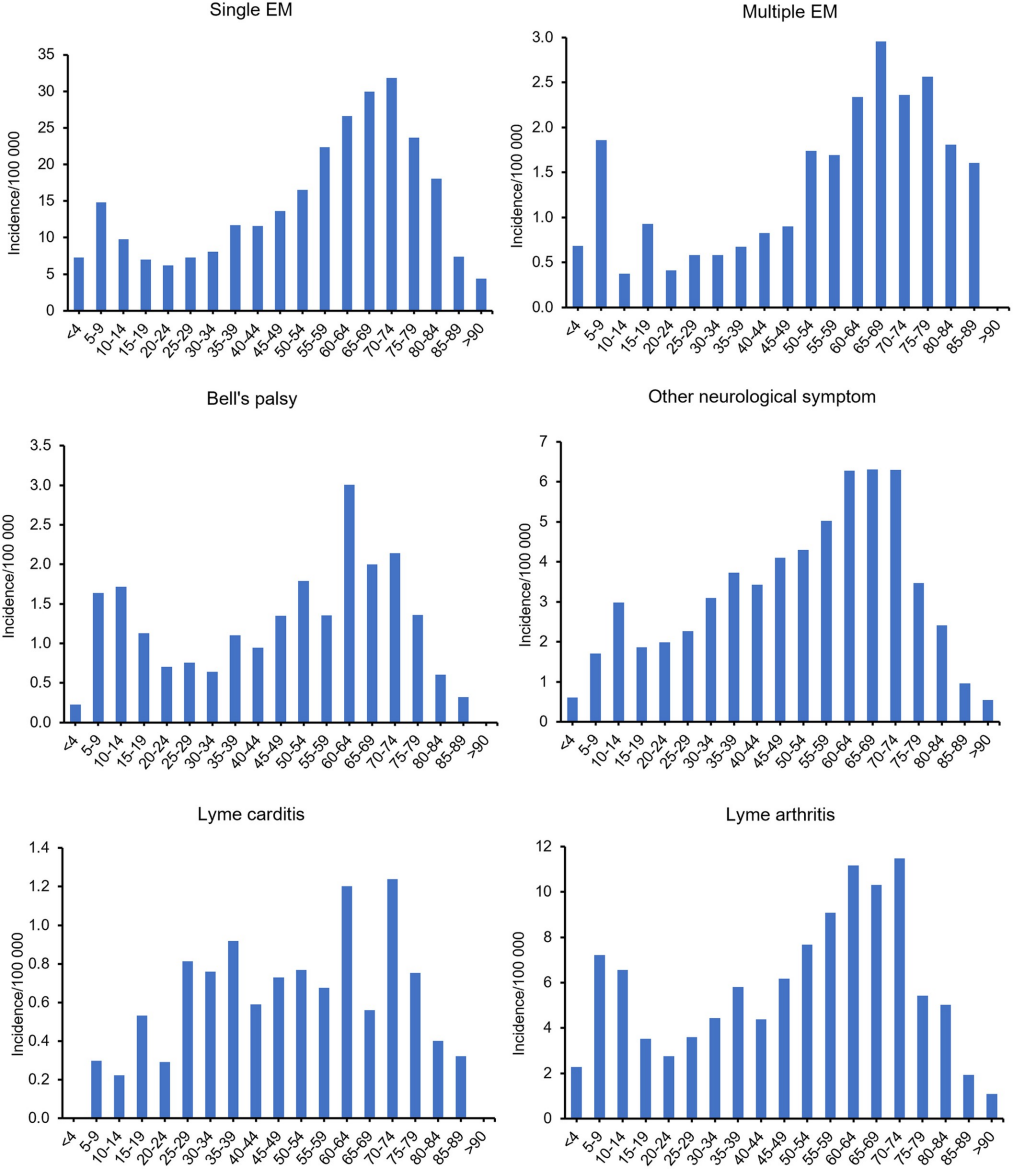
Incidence per 100 000 population of clinical manifestations reported for Lyme disease cases, Canada, 2009–2019. Symptoms reported for single erythema migrans (EM) (n = 3,583), multiple EM (n = 309), Bell’s palsy (n = 320), other neurological symptoms (n = 858), Lyme carditis (n = 156), Lyme arthritis (n = 1,510). Age was missing for cases reporting single EM (n = 5) and Lyme arthritis (n = 1).

Clinical manifestations presented mostly in a bimodal distribution, with a peak in childhood followed by a decline in early adulthood before the largest peak in later adulthood. Incidence of disease stage followed similar trends (S1 Fig). For single EM and multiple EM, peaks in childhood were between ages 5–9, while peaks in adults were between ages 55–79 and 60–79, respectively (Fig 2). Bell’s palsy incidence peaked in children aged 5–14 and in adults aged 60–64. Lyme arthritis peaked in children aged 5–14 and in adults aged 60–74. Of note, the incidence in many of the adult age categories exceeded the incidence in children aged 5–9 or 5–14.

Incidence of reported other neurological manifestations peaked in children aged 10–14 and adults aged 55–74. After decreased incidence in adolescence and early adulthood, incidence steadily increased so that, beginning at age 30–34, incidence was higher than the peak observed in children aged 10–14.

Cardiac symptoms were more evenly distributed across age groups, with lower incidence in children and spikes in incidence in adults aged 25–39, 60–64 and 70–74.

### Geographic distribution

**Fig 3:**
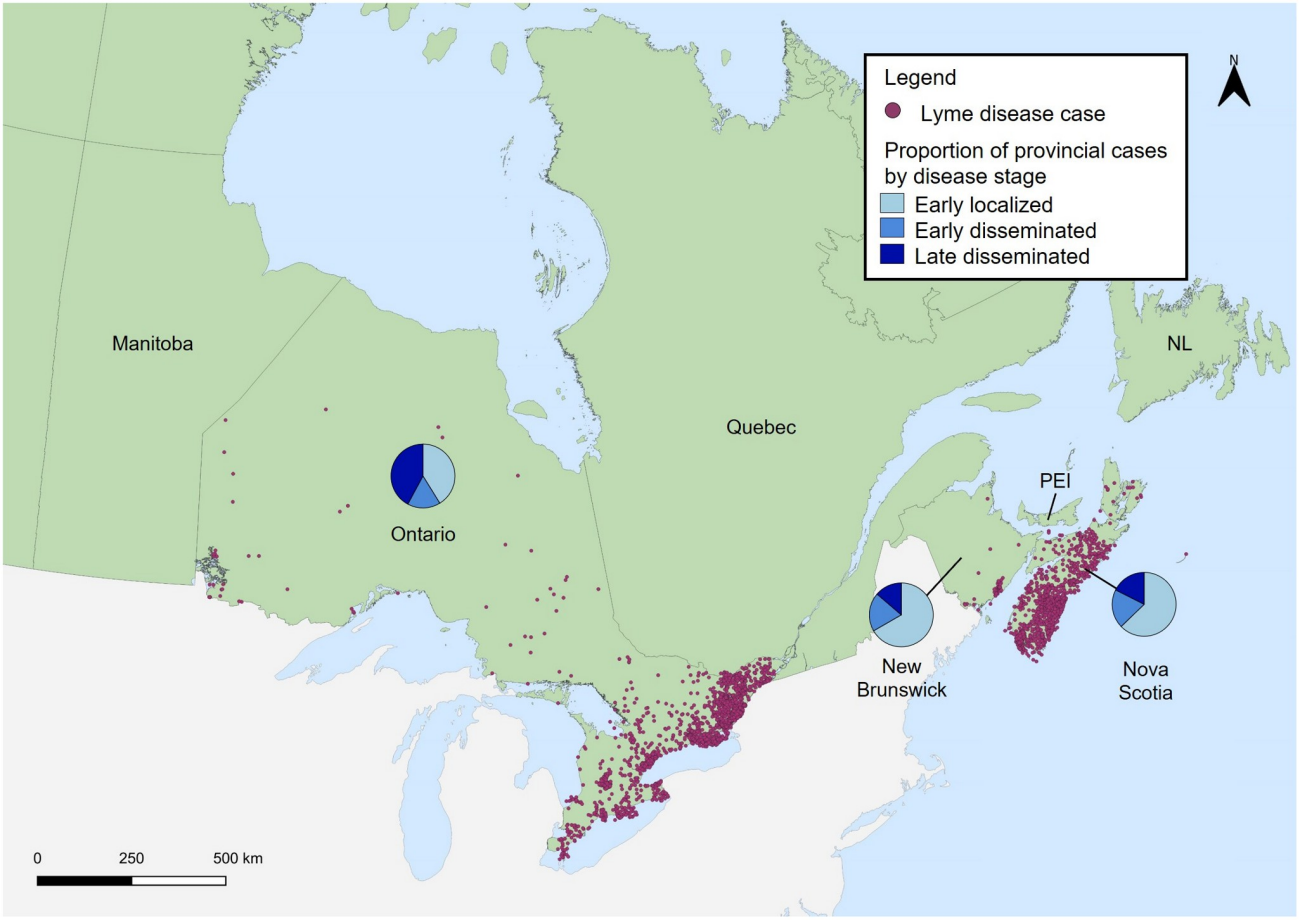
Geographic distribution of Lyme disease cases and proportion of cases by disease stage, Canada, 2009–2019*. * Each dot represents the probable location of acquisition randomly distributed at the census subdivision level (n = 4,036) or the forward sortation area of residency for cases from Nova Scotia in 2019 (n = 373). The entire province of Nova Scotia was declared a LD risk area in 2018, so residency was inferred as the probable location of acquisition in the absence of travel history. Disease stage was available for 4,419 cases. The data on clinical manifestations are provided by provinces that are participating in the LDES and included in this analysis (British Columbia, Alberta, Ontario, New Brunswick, Nova Scotia, Newfoundland & Labrador [NL] and Prince Edward Island [PEI]). Manitoba participates in the LDES but was excluded from this analysis. Saskatchewan, Quebec and the Territories do not submit data to the LDES; instead, they provide limited information through the CNDSS. All cases reported by Alberta and Prince Edward Island were travel-related cases and thus not included on the map. All cases reported by British Columbia were either travel-related cases or had no location of acquisition at the sub-provincial level. No cases from Newfoundland & Labrador met inclusion criteria for the study.

The majority of cases in New Brunswick (66.6%; 34/51) and Nova Scotia (62.7%; 1,155/1,840) exhibited clinical signs that aligned with early localized LD (Fig 3). In contrast, more cases in Ontario were reported with late disseminated LD (42.1%; 1,065/2,528) than early localized (41.2%; 1,041/2,528). All cases reported by Alberta (n = 30) and Prince Edward Island (n = 11), and the majority of cases reported by British Columbia (77.8%; 7/9) were travel related. No cases from Newfoundland & Labrador met inclusion criteria for the study.

### Changes over time

There was an increase in reported cases acquired in Canada with at least one clinical manifestation before and after the updated case definition: 1,367 between 2009 and 2015 and 3,334 between 2016 and 2019 (Table 2). The average number of cases increased from 195.3 cases per year from 2009–2015 to 833.5 cases per year from 2016–2019.

Patients reporting clinical manifestations in 2016–2019 were older than those reporting in 2009–2015 (54 vs 51 years; p<0.001) (Table 2). Reports of other neurological manifestations increased from 2009–2015 (14.6%, 95% CI: 12.7–16.5) to 2016–2019 (19.8%, 95% CI: 18.4–21.2). Reports of Lyme arthritis decreased from 2009–2015 (35.8%, 95% CI: 33.3–38.5) to 2016–2019 (30.7%, 95% CI: 29.1–32.3).

With regards to the disease stage, the proportion of late disseminated LD cases decreased from 2009–2015 (35.9%, 95% CI: 33.3–38.5) to 2016–2019 (30.6%, 95% CI: 29.1–32.2) (Table 2). The distribution of probable and confirmed cases did not significantly change across periods.

Mean annual incidence increased in all regions between 2009–2015 and 2016–2019, but markedly in the Atlantic region (Table 3). In the multivariate analyses, cases from the central region had lower odds of being early localized LD (odds ratio [OR]: 0.4, 95% CI: 0.4–0.5) and early disseminated LD (OR: 0.8, 95% CI: 0.7–1.0) but higher odds of late disseminated LD (OR: 3.5, 95% CI: 3.0–4.0) compared to the reference Atlantic region (Table 4).

**Table 3:** Mean annual incidence rate of Lyme disease cases by region, Canada, 2009–2019*.

Region **	Mean annual incidence rate per 100,000				P value
	All years	2009–2015	2016–2019	Difference (95% CI)	
Central	1.7	0.8	3.1	2.3 (0.2–4.4)	0.039
Atlantic	10.1	4.6	19.8	15.2 (5.9–24.6)	0.009
Total ***	2.6	1.2	4.9	3.7 (0.8–6.6)	0.023

Abbreviation: CI, confidence interval

* n = 4,461

** Only LD cases that were acquired within the same province in which they were reported were included

*** Only the provinces included in the analysis were used for the total population denominator

**Table 4:** Multivariate logistic regression models predicting occurrence of Lyme disease stage from the Lyme Disease Enhanced Surveillance system, Canada, 2009–2019*.

Explanatory variables **	Estimate	Standard error	Odds ratio	95% CI	Wald test	*P* value
**Early localized (erythema migrans)**						
Central vs Atlantic	-0.9	0.1	0.4	0.4–0.5	-13.8	<0.001
10–19 years vs 0–9 years	-0.4	0.2	0.6	0.5–0.9	-2.9	0.004
20–29 years vs 0–9 years	-0.3	0.2	0.7	0.5–1.0	-2.1	0.039
30–39 years vs 0–9 years	-0.4	0.1	0.7	0.5–0.9	-2.8	0.005
Mar–May vs Dec–Feb	0.8	0.2	2.2	1.4–3.6	3.4	<0.001
Jun–Aug vs Dec–Feb	0.7	0.2	2.1	1.4–3.2	3.5	<0.001
Sep–Nov vs Dec–Feb	0.6	0.2	1.8	1.2–2.8	2.6	0.008
2016–2019 vs 2009–2015	0.2	0.1	1.2	1.1–1.4	2.8	0.005
**Early disseminated (neurologic and cardiac symptoms; multiple erythema migrans)**						
Central vs Atlantic	-0.2	0.1	0.8	0.7–1.0	-2.5	0.011
Jun–Aug vs Dec–Feb	0.8	0.3	2.3	1.2–4.4	2.5	0.013
**Late disseminated (arthritis)**						
Central vs Atlantic	1.2	0.1	3.5	3.0–4.0	16.8	<0.001
80+ years vs 0–9 years	-0.5	0.2	0.6	0.4–1.0	-2.1	0.041
Mar–May vs Dec–Feb	-1.0	0.2	0.4	0.2–0.6	-4.0	<0.001
Jun–Aug vs Dec–Feb	-1.3	0.2	0.3	0.2–0.4	-5.9	<0.001
Sep–Nov vs Dec–Feb	-0.9	0.2	0.4	0.3–0.6	-4.0	<0.001
2016–2019 vs 2009–2015	-0.3	0.1	0.8	0.7–0.9	-3.5	<0.001

Abbreviation: CI, confidence interval

* n = 4,450

** Only LD cases that were acquired within the same province in which they were reported were included

Cases reported from March through November had higher odds of being early localized LD (March-May OR: 2.2, 95% CI: 1.4–3.6; June-August OR: 2.1, 95% CI: 1.4–3.2; September-November OR: 1.8, 95% CI: 1.2–2.8) but lower odds of being late disseminated LD (March-May OR: 0.4, 95% CI: 0.2–0.6; June-August OR: 0.3, 95% CI: 0.2–0.4; September-November OR: 0.4, 95% CI: 0.3–0.6) compared to cases reported from December through February. Cases reported from June through August had higher odds of being early disseminated than those reported from December through February (OR: 2.3, 95% CI: 1.2–4.4).

The odds of age groups 10–19, 20–29 and 30–39 years reporting early localized LD were 0.6 (95% CI: 0.5–0.9), 0.7 (95% CI: 0.5–1.0) and 0.7 (95% CI: 0.5–0.9), respectively, less than the reference group of 0–9 years old. Meanwhile cases over the age of 80 were less likely to be reported as late disseminated LD compared to those between 0–9 years old (OR: 0.6, 95% CI: 0.4–1.0).

Cases reported in 2016–2019 had higher odds of being early localized LD (OR: 1.2, 95% CI: 1.1–1.4) but lower odds of being late disseminated LD (OR: 0.8, 95% CI: 0.7–0.9).

### Seasonality

**Fig 4:**
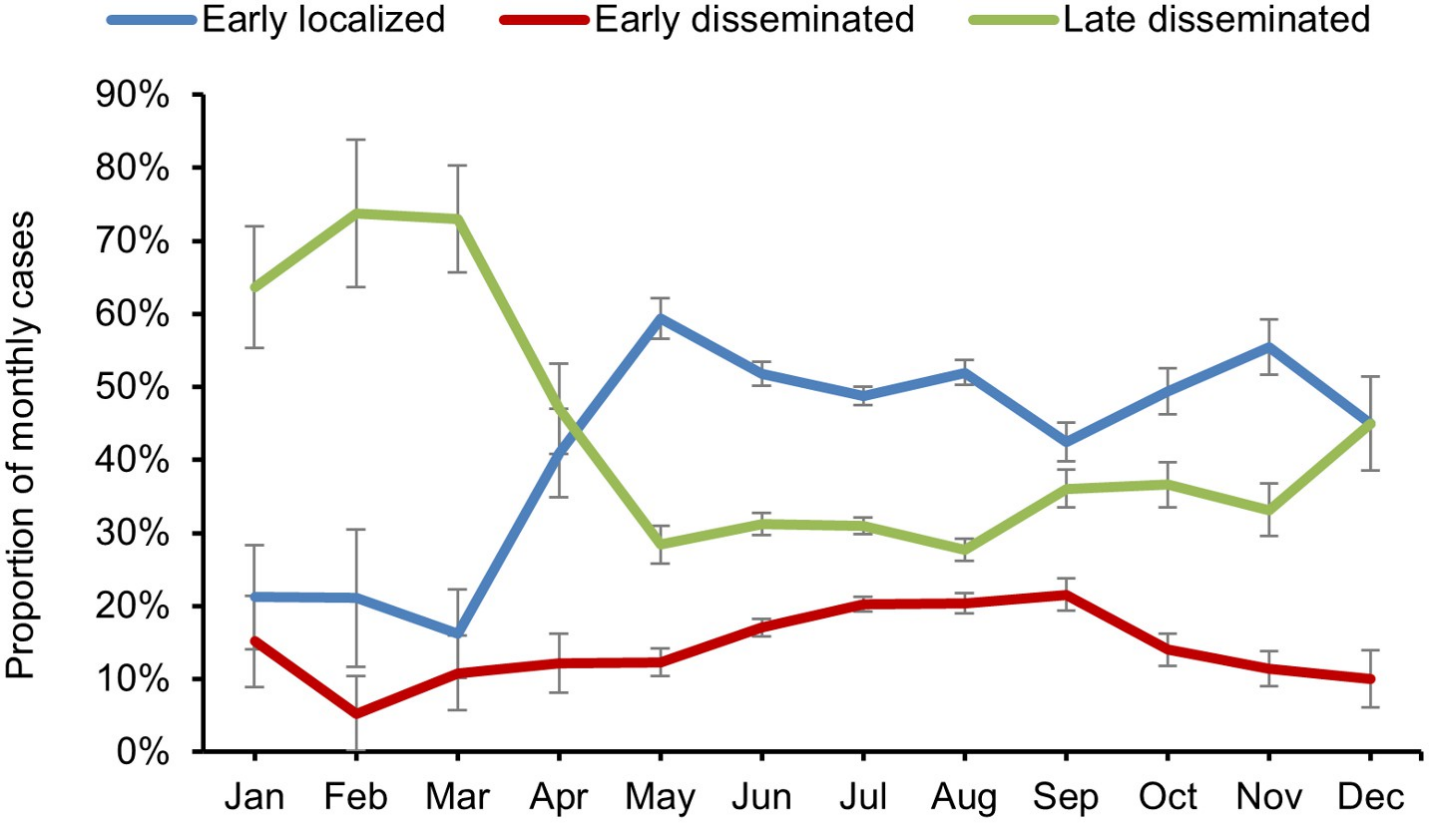
Proportions of Lyme disease cases by month and by disease stage, Canada, 2009–2019. Early localized cases (n = 2,339) were characterized as those presenting with only single erythema migrans (EM). Early disseminated cases (n = 851) were characterized as those reporting at least one of the following signs, with or without single EM: multiple EM, Bell’s palsy, other neurological signs, or cardiac signs. Late disseminated cases (n = 1,511) were characterized as any case reporting arthritis, with or without reporting any other sign or symptom. Error bars represent standard deviation.

Month of illness onset was reported for all 4,701 cases. Early localized and early disseminated LD cases were reported mainly during the spring, summer, and fall months, from May through November (Fig 4). Comparatively, late disseminated LD cases were reported more often in the winter from January through March.

## Discussion

This report provides an epidemiologic analysis of the clinical manifestations of LD cases acquired in Canada from seven provinces reporting to the LDES between 2009 and 2019. There was a significant decrease in the proportion of late disseminated LD cases reported after 2016.

The clinical presentation of LD from the LDES is similar to those presenting in the United States. A single EM was the most common presentation of LD (76.4%), which is similar to estimates from the United States, where 70% to 80% of symptomatic individuals experienced this clinical sign [19].

With respect to age, the incidence of patients presenting several clinical manifestations showed a bimodal distribution. An initial peak is observed in children and an additional peak is observed in adults aged between 55–75 years old. This result is consistent with findings in the United States [4, 20]. Notably, cardiac signs were observed to be higher in proportion in those aged 25–39, 60–64 and 70–74. This pattern is similar to previous literature on reported Lyme carditis from the United States. A study using national surveillance data showed two peaks within patients presenting Lyme carditis, those 20–39 years of age and ≥75 years of age [20]. Similar to reports in the United States, fatal cardiac manifestations are rare [7]. There has been one report of a death of a 37-year-old due to Lyme carditis in Manitoba [21].

The Central region had higher odds of late disseminated LD and lower odds of early localized and early disseminated LD compared to the Atlantic region. These observed differences in LD disease stage across Canada may be due to several factors. The range of the *Ixodes* tick has expanded over time, and LD risk areas within and between provinces differ substantially. LD surveillance aims to identify risk areas where public health messaging may be targeted. Due to competing public health priorities, possible challenges in resourcing tick surveillance in some jurisdictions may limit timely identification of emerging risk areas. Therefore, related actions such as seeking medical attention and advice or even consideration of LD as a primary diagnostic option by physicians may be hindered, potentially delaying diagnosis in these areas. Over the last two decades, the number of *I*. *scapularis* submitted to the National Microbiology Laboratory of PHAC through passive surveillance has increased (S2 Fig). Populations of *I*. *scapularis* infected with LD bacteria have quickly spread their scope and geographical range northward in central and eastern provinces like Manitoba, Ontario, Quebec, New Brunswick and Nova Scotia where the tick has been established and endemic for several years [22–30]. However, in the prairie provinces of Saskatchewan and Alberta, only adventitious blacklegged ticks brought in by migratory hosts have been detected through passive surveillance; no established *I*. *scapularis* tick populations have been detected so far [31, 32]. In British Columbia, although populations of *I*. *pacificus* ticks that transmit LD bacteria in this province have been established for several years, the geographic scope has remained stable, and the prevalence of infection has remained relatively low and stable over the years [33, 34].

In addition, previous studies have shown that there are different strains of *B*. *burgdorferi* circulating in Canada, with some showing geographic patterns of distribution [35]. It has been suggested that strain-type may influence disease severity and diagnostics [36–39]. However, how strains circulating in Canada impact these factors is an ongoing area of research.

Incidence of LD cases reported to the LDES increased from 2009 to 2019, mirroring the increase of total LD cases reported in Canada over the same period [12]. Vector tick populations have been increasing in size and scope across Canada [40], leading to an increase in LD risk areas and greater exposure to ticks infected with *B*. *burgdorferi*. This may be due to changes in climate that influence the occurrence and abundance of ticks and the transmission of tick-borne pathogens by diverse effects on tick and pathogen lifecycles [41].

We observed an increase in the average number and mean annual incidence of LD cases reported per year in the 2016–2019 period as compared to the 2009–2015 period across all regions, but especially in the Atlantic region. Several factors may explain this discrepancy. The 2016 case definition [16] enables provinces to identify LD risk areas in a less resource intensive, and a more timely and standardized manner, allowing more cases to be reported. It also allows some provinces to report both confirmed and probable cases when previously they were not able to distinguish between the two.

Additionally, awareness of the public and healthcare professionals may also have contributed to a greater increase in LD cases during the second period. A national study showed a significant increase in awareness of LD [42] after the launch of a national communication campaign in 2014 [43]. This national awareness may also explain the decrease in the proportion of cases presenting as late disseminated LD during the 2016–2019 period as compared to the 2009–2015 period. The accurate diagnosis and appropriate management of patients bitten by ticks or presenting LD symptoms are key factors for reducing the impact of the disease [44]. While there was a decrease in the proportion of cases presenting as late disseminated LD, more than 30% of individuals still presented at this stage during the 2016–2019 period. Continued efforts should be made to increase awareness so that infections are identified and treated early.

The seasonal patterns observed in this study align with the observed seasonal patterns in local jurisdictions in Canada [12] and the United States [20]. Individuals are at risk of tick bites and LD infection from the spring through the fall months when the weather conditions (e.g., temperature and humidity) are most suitable for tick activity. The spring and summer months are when the activity of nymphs is highest, which are most implicated in LD transmission, and individuals are commonly outdoors [45–47]. In Canada, nymphs have been shown to be active from April to late September, whereas adults were active in two peaks: newly molted adults being active from late September to early December, with those that failed to find a host at this time being active from March through June of the following year [48, 49]. Several studies in Canada have demonstrated that climate features (e.g., temperature and rainfall) influence the establishment of the blacklegged tick and impact the development of reproducing populations [29, 40, 50, 51]. A recent study in the province of Québec showed that more ticks were found in active field surveillance when temperature and relative humidity were higher [52]. The accumulation of degree days above threshold temperatures also contributes to the reproduction of tick populations [52, 53]. In the United States, studies have demonstrated that relative humidity and temperature influence questing height and behavior of ticks [54–56].

It is not surprising that LD presenting as late disseminated occurred at a higher proportion in December through April, as this stage represents individuals who had progressive infection after being missed at early stage of infection [3, 8].

### Limitations

There are limitations to this analysis. These findings are not generalizable to all LD cases in Canada but are only descriptive of the cases submitted through the LDES which were acquired in Canada with data available on clinical manifestations. The data for this study were extracted from a surveillance system that is passive in nature. Provincial health authorities report data on a voluntary basis, meaning case numbers may be incomplete or lacking full clinical manifestation information. Under reporting is a challenge for any surveillance system [57, 58]. Some patients may not recognize early infection and seek medical attention, and while positive laboratory results are reported routinely to local public health authorities, early localized infection lacks laboratory confirmation. As such, knowledge of these cases is dependent upon the primary care provider reporting to local public health authorities, likely leading to under reporting of these cases [59]. On this note, attention should be brought to the fact that some provinces do not report clinical signs to the LDES system, and therefore an unknown number of LD cases are absent from this national analysis. Additionally, since reporting clinical signs is voluntary, misclassification may occur when determining LD stage for a case.

## Conclusion

The results from this analysis describe the clinical manifestations associated with Canadian LD cases from the LDES system and show trends of LD diagnosis in several Canadian provinces over time. Decreases in reports of late disseminated LD potentially reflect improvements in LD identification and treatment. Continued education and awareness raising efforts for the public and clinicians would improve the consideration of LD in the appropriate circumstances. Future research efforts should explore the relationship between age and clinical manifestations, and the representativeness of cases reported to the LDES system to all LD cases in Canada. Observations from this analysis can further guide public health authorities to provide targeted prevention strategies and act as a resource for health workers for early diagnosis of LD patients. Continued surveillance and public health preventative interventions will be beneficial in reducing the risk of LD for Canadians.

## Supporting information

S1 Fig Incidence per 100 000 population of disease stage for Lyme disease reported cases, Canada, 2009–2019. Age was reported for early localized (n = 2,335), early disseminated (n = 851), and late disseminated (n = 1,510) Lyme disease cases. Age was missing for early localized (n = 4) and late disseminated (n = 1) cases.
(TIF)

S2 Fig Number of Ixodes scapularis submitted to the National Microbiology Laboratory of through passive surveillance, 2009–2019. Ticks (n = 85,400) were submitted to the National Microbiology Laboratory (NML) of the Public Health Agency of Canada from individuals or public health laboratories. Ticks submitted to passive surveillance programs in provinces that do not forward ticks to NML are not included (though ticks from these provinces that were submitted directly to the NML for tick identification and pathogen testing are included). Submission requirements for passive tick surveillance programs have changed over time, which has affected the quantity of ticks submitted in certain jurisdictions.
(TIF)
