# Supplementary figures and images for: Epidemiology and clinical manifestations of reported Lyme disease cases: Data from the Canadian Lyme disease enhanced surveillance system
Source: PLoS One. 2023 Dec 15;18(12):e0295909. doi: 10.1371/journal.pone.0295909 (PMC10723709; doi:10.1371/journal.pone.0295909)

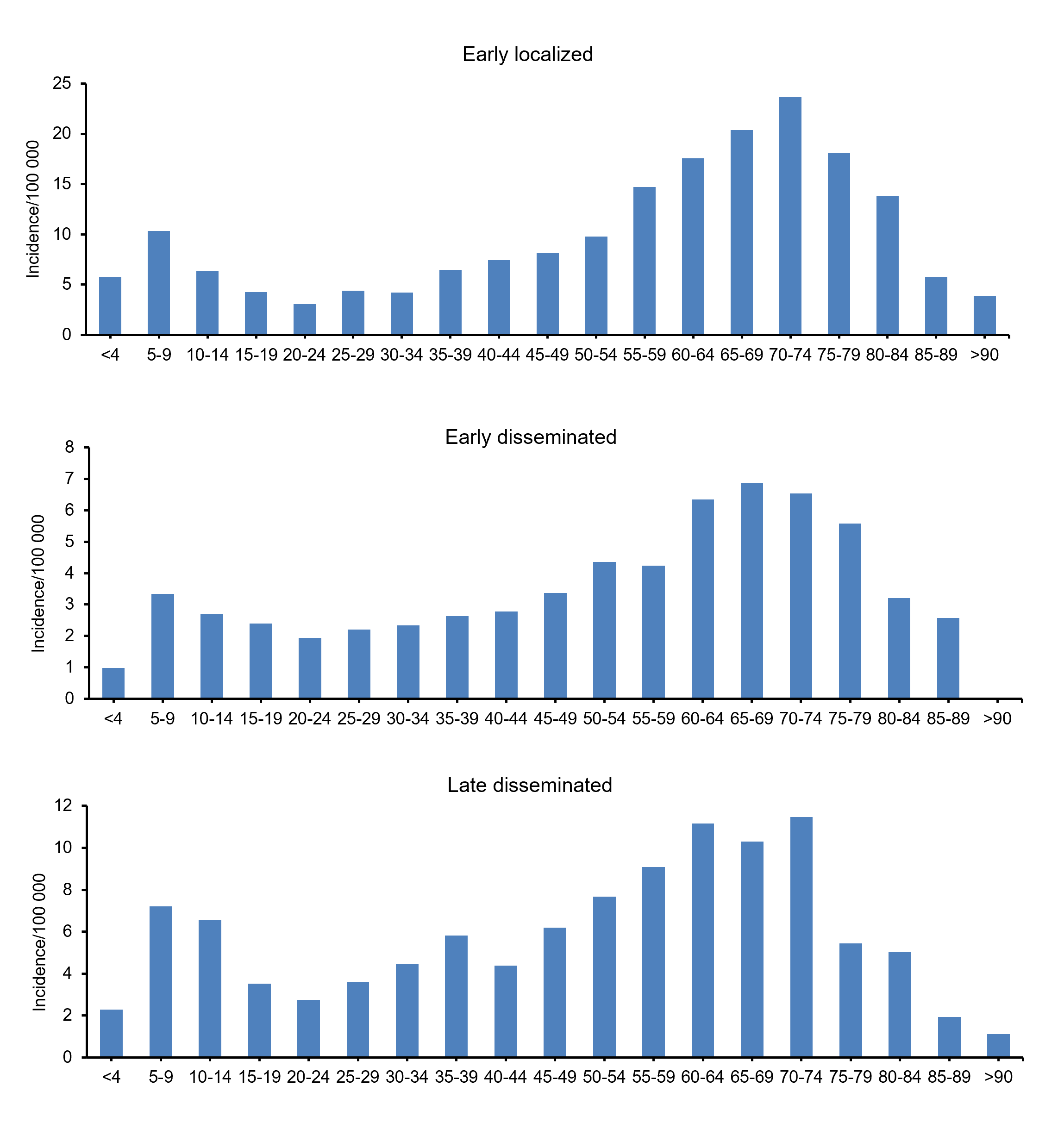

Supplement: S1 Fig — Age was reported for early localized (n = 2,335), early disseminated (n = 851), and late disseminated (n = 1,510) Lyme disease cases. Age was missing for early localized (n = 4) and late disseminated (n = 1) cases. (TIF) [file pone.0295909.s001.tif]

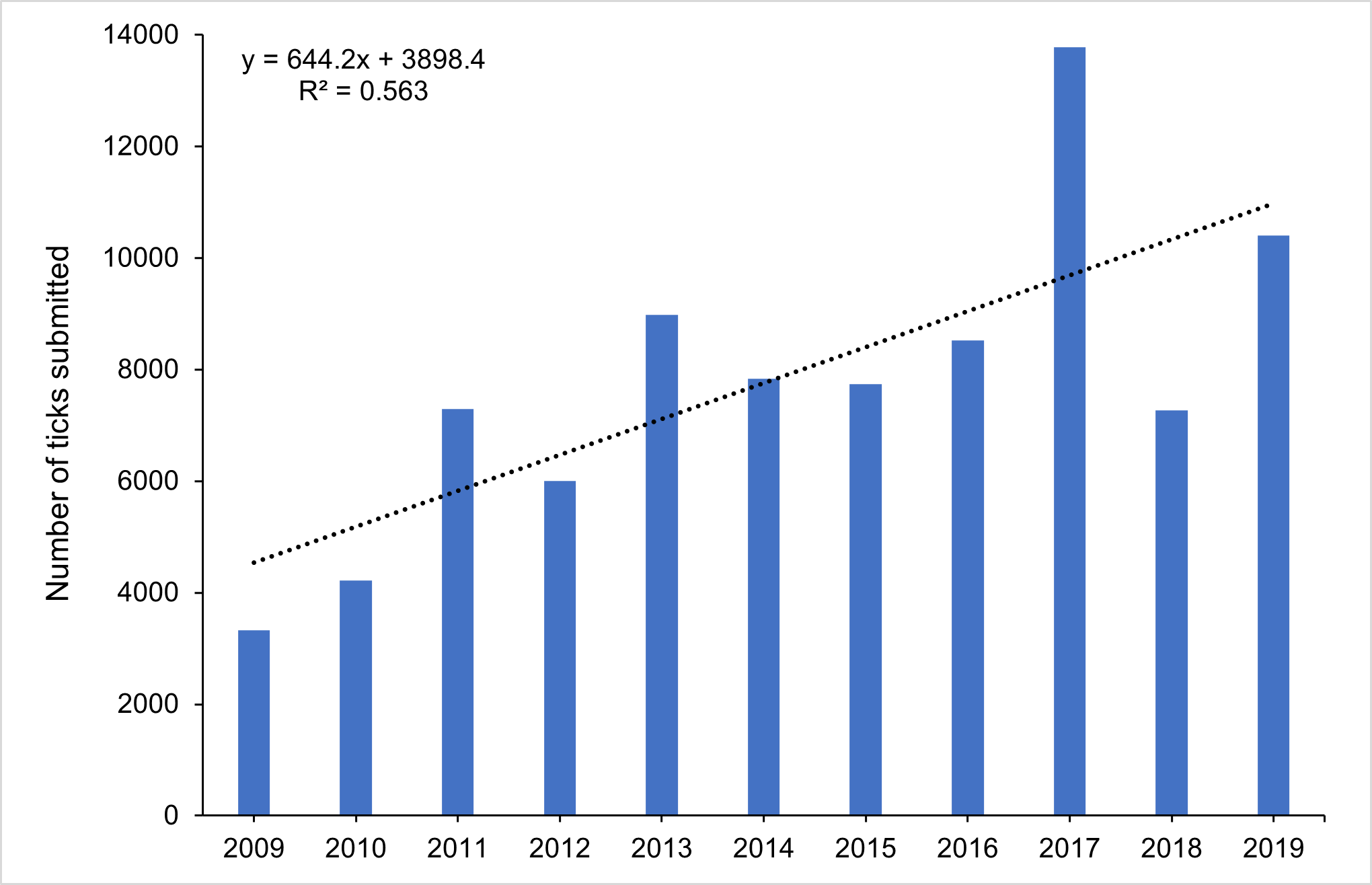

Supplement: S2 Fig — Ticks (n = 85,400) were submitted to the National Microbiology Laboratory (NML) of the Public Health Agency of Canada from individuals or public health laboratories. Ticks submitted to passive surveillance programs in provinces that do not forward ticks to NML are not included (though ticks from these provinces that were submitted directly to the NML for tick identification and pathogen testing are included). Submission requirements for passive tick surveillance programs have changed over time, which has affected the quantity of ticks submitted in certain jurisdictions. (TIF) [file pone.0295909.s002.tif]
